# Open-label phase IV trial evaluating nusinersen after onasemnogene abeparvovec in children with spinal muscular atrophy

**DOI:** 10.1172/JCI193956

**Published:** 2025-09-16

**Authors:** Crystal M. Proud, Richard S. Finkel, Julie A. Parsons, Riccardo Masson, John F. Brandsema, Nancy L. Kuntz, Richard Foster, Wenjing Li, Ross Littauer, Jihee Sohn, Stephanie Fradette, Bora Youn, Angela D. Paradis, on behalf of the RESPOND Study Group

**Affiliations:** 1 Neurology and Neuromuscular Medicine, Children’s Hospital of The King’s Daughters, Norfolk, Virginia, USA.; 2 Center for Experimental Neurotherapeutics, Department of Pediatric Medicine, St. Jude Children’s Research Hospital, Memphis, Tennessee, USA.; 3 Department of Pediatrics, Children’s Hospital Colorado, University of Colorado School of Medicine, Aurora, Colorado, USA.; 4 Neuroimmunology and Neuromuscular Disease Unit, Fondazione IRCCS Istituto Neurologico Carlo Besta, Milan, Italy.; 5 Division of Neurology, Children’s Hospital of Philadelphia, Philadelphia, Pennsylvania, USA.; 6 Division of Neurology, Ann & Robert H. Lurie Children’s Hospital of Chicago, Chicago, Illinois, USA.; 7 Biogen, Maidenhead, United Kingdom.; 8 Biogen, Cambridge, Massachusetts, USA.; 9 The RESPOND Study Group is detailed in Supplemental Methods.

**Keywords:** Clinical Research, Muscle biology, Gene therapy

## Abstract

**BACKGROUND:**

Spinal muscular atrophy (SMA) is a rare genetic neuromuscular disease caused by deletions or mutations of the survival motor neuron 1 (*SMN1*) gene. Despite the availability of genetically based treatments for SMA, functional impairments and weakness persist in treated symptomatic individuals. This study addresses whether additional treatment after gene transfer therapy could provide further clinical benefits.

**METHODS:**

Interim day 302 findings are described from the phase IV open-label RESPOND trial evaluating nusinersen in participants aged ≤36 months who had suboptimal clinical status following onasemnogene abeparvovec (OA) treatment, as determined by the investigator.

**RESULTS:**

Thirty-seven participants included in the interim analysis were symptomatic at the time of OA administration. Most (92%) had 2 *SMN2* gene copies. Age at first nusinersen dose (median) was 9.1 (range, 3–33) months for participants with 2 *SMN2* copies and 34.2 (range, 31–36) months for those with 3 *SMN2* copies, while time from OA dose to first nusinersen dose (median) was 6.3 (range, 3–31) and 13.3 (range, 10–22) months, respectively. Participants had elevated neurofilament light chain (NfL) levels and low compound muscle action potential (CMAP) amplitudes at baseline, suggesting active neurodegeneration and severe denervation at study entry. Improvements from baseline were observed across a range of outcomes on day 302, including motor function (HINE-2 and CHOP-INTEND total score), achievement of independent sitting, NfL levels, CMAP, and investigator- and caregiver-reported outcomes. Mean NfL levels decreased rapidly from baseline to day 183 and remained low on day 302. Mean ulnar and peroneal CMAP amplitudes increased. No safety concerns were identified.

**CONCLUSION:**

Improvements in clinical and biomarker outcomes support the benefit of nusinersen treatment in infants and children with suboptimal clinical status following OA.

**TRIAL REGISTRATION:**

ClinicalTrials.gov NCT04488133; EudraCT 2020-003492-18.

**FUNDING:**

This study was sponsored by Biogen.

## Abstract 2

Improvements in clinical and biomarker outcomes were observed after nusinersen treatment in children with spinal muscular atrophy who had suboptimal clinical status following onasemnogene abeparvovec.

## Introduction

Spinal muscular atrophy (SMA) is an autosomal-recessive neuromuscular disease characterized by progressive neurodegeneration, muscle atrophy, and muscle weakness (1–3). It is caused by deletions or mutations of the survival motor neuron 1 (*SMN1*) gene, reducing/stopping production of full-length SMN protein. The paralogous *SMN2* gene produces mostly truncated, dysfunctional SMN protein, with only approximately 10% of *SMN2* transcripts resulting in the production of full-length SMN protein (1–3). SMN protein insufficiency leads to motor neuron degeneration and declining motor, bulbar, and respiratory function (3). Greater *SMN2* copy numbers are generally associated with increased functional SMN protein and thus correlate with a milder SMA phenotype. Individuals with 2 *SMN2* copies typically develop a more severe form of the disease, with symptoms readily observable within a few weeks to months after birth (4).

The approval of disease-modifying treatments (DMTs) over the past decade has notably improved the disease trajectories and outcomes for individuals with SMA. Nusinersen, the first DMT approved for SMA in the US, is an antisense oligonucleotide (ASO) administered intrathecally that modifies the splicing of *SMN2* precursor mRNA to increase levels of full-length SMN protein (5–7). Nusinersen has demonstrated clinically meaningful and sustained efficacy in a range of presymptomatic and symptomatic individuals across the SMA spectrum, with a well-established safety profile from over 10 years in clinical trials and postmarketing settings (8–11). The current approved dose for nusinersen in the US is four 12 mg loading doses (first 3 loading doses at 14-day intervals; fourth loading dose 30 days after the third dose), followed by 12 mg maintenance doses every 4 months (7).

Onasemnogene abeparvovec (OA) is a one-time gene transfer therapy delivered intravenously. It uses an adeno-associated virus serotype 9 (AAV9) vector to deliver a copy of the human *SMN* gene (12–14). Although OA therapy substantially improves survival and motor function, challenges such as motor delays or residual weakness may persist even with prompt OA treatment, particularly in individuals with 2 *SMN2* copies (12, 15, 16).

Preclinical animal models and limited human postmortem studies suggest that the AAV9 vector transduces only a subset of motor neurons (17–19). Motor neurons that are not transduced remain subject to the natural progression of this degenerative disease, which would be expected to impact clinical outcomes over time (20–22). This raises the question of whether nusinersen-driven increases in SMN protein in untransduced motor neurons could contribute to broader target engagement and provide additional clinical benefits for individuals with SMA. However, due to the lack of clinical trials on the combined use of genetically based therapies to date, there are limited data to guide treatment decisions for optimizing clinical outcomes in SMA.

RESPOND (ClinicalTrials.gov NCT04488133) (23) is an ongoing study evaluating the clinical outcomes and safety of nusinersen administration in participants with SMA previously treated with OA. The objective of this interim analysis is to provide clinical, biomarker, and safety findings for RESPOND participants on day 302 after nusinersen initiation.

## Results

### Participants.

**Figure 1:**
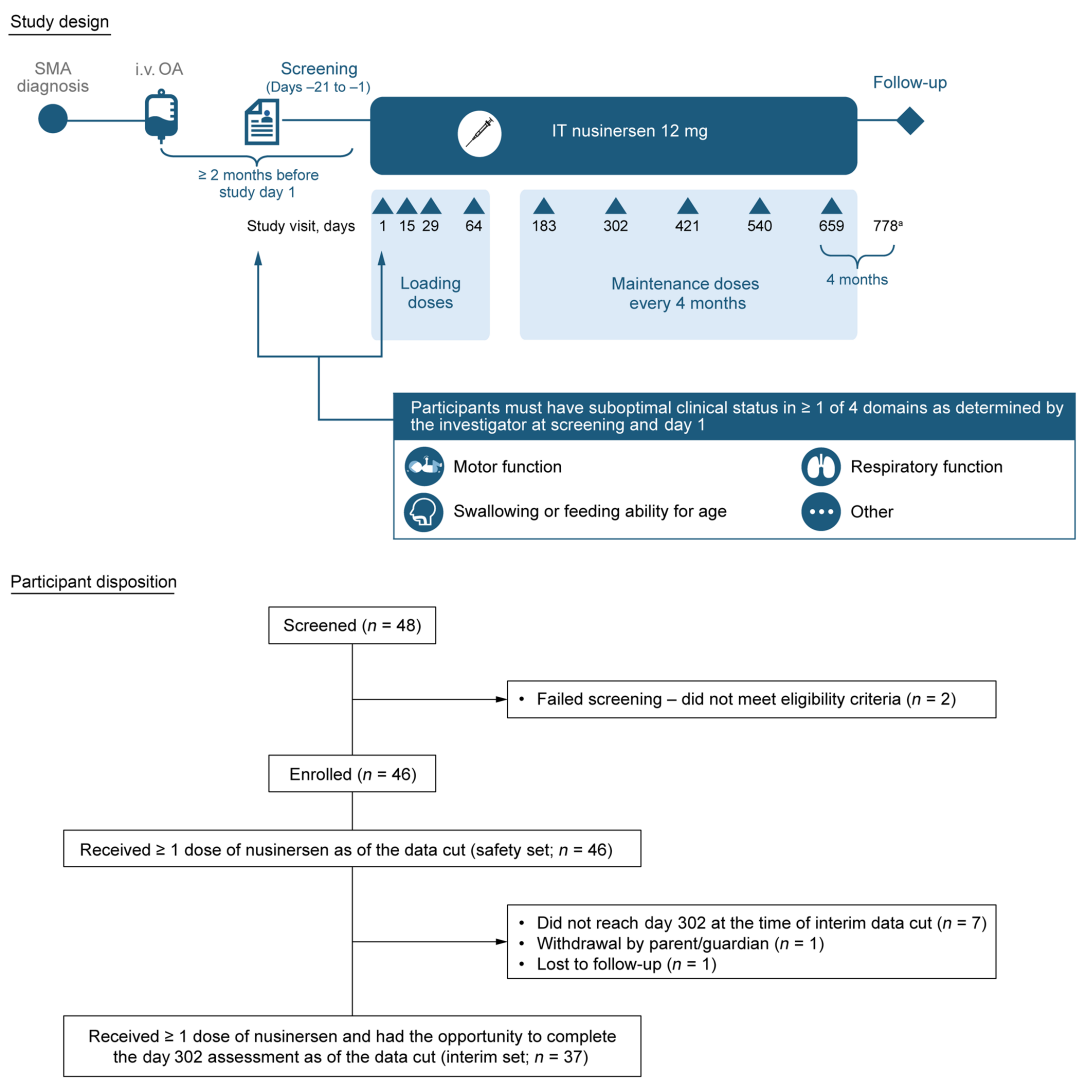
Study design and participant disposition. ^a^Or 4 months from last dose. IT, intrathecal.

In total, 46 individuals were enrolled (Figure 1). The efficacy set included 37 participants who had received ≥1 dose of nusinersen and had the opportunity to complete the day 302 assessment. The safety set included all 46 enrolled participants.

### Baseline demographics and clinical characteristics.

Of the 37 participants in the efficacy set, 34 (92%) had 2 *SMN2* copies, and 3 (8%) had 3 *SMN2* copies (Table 1). Among participants with 2 *SMN2* copies, 21 were aged ≤9 months at first nusinersen dose and 13 were aged >9 months (Supplemental Table 1; supplemental material available online with this article; https://doi.org/10.1172/JCI193956DS1).

Age at first nusinersen dose and time from OA dose to first nusinersen dose varied greatly among participants (Table 1 and Supplemental Table 1). Median age at first nusinersen dose was 9.1 (range, 3–33) months for those with 2 *SMN2* copies and 34.2 (range, 31–36) months for those with 3 *SMN2* copies. Median time from OA dose to first nusinersen dose was 6.3 (range, 3–31) months and 13.3 (range, 10–22) months, respectively.

Most of the 37 participants had multiple investigator-identified domains with suboptimal clinical status; 30 (88%) in the 2-*SMN2*-copy group and 1 (33%) in the 3-*SMN2*-copy group, respectively. Suboptimal clinical status in motor function was most commonly reported, with 94% in the 2-*SMN2*-copy group and 100% in the 3-*SMN2*-copy group. Suboptimal clinical status in swallowing or feeding ability for age and respiratory function was reported in 59% and 71% in the 2-*SMN2*-copy group, and 33% and 0% in the 3-*SMN2*-copy group. Detailed descriptions of suboptimal clinical status provided by investigators are summarized in Supplemental Table 2.

The majority of participants with 2 *SMN2* copies (27 [79%]) were unable to sit without support at baseline (typical window for sitting achievement in healthy children: 3.8–9.2 months) (24). All 3 participants with 3 *SMN2* copies were able to sit without support, but none were able to walk without support (typical window for walking achievement in healthy children: 8.2–17.6 months) (24). Penetration and aspiration of thin liquids on swallowing were observed in 26% and 33% of participants with 2 *SMN2* copies, respectively.

For ulnar compound muscle action potential (CMAP) amplitude, 27 (79%) participants in the 2-*SMN2*-copy group and 1 (33%) in the 3-*SMN2*-copy group had a baseline value of ≤1 mV. Additionally, 32 (94%) and 1 (33%), respectively, had a baseline value of ≤2 mV. For peroneal CMAP amplitude, 18 (58%) participants in the 2-*SMN2*-copy group and 0 (0%) in the 3-*SMN2*-copy group had a baseline value of ≤1 mV. Twenty-nine (94%) and 2 (67%) participants, respectively, had a baseline value of ≤2 mV.

### Changes in motor function and motor milestones.

**Figure 2:**
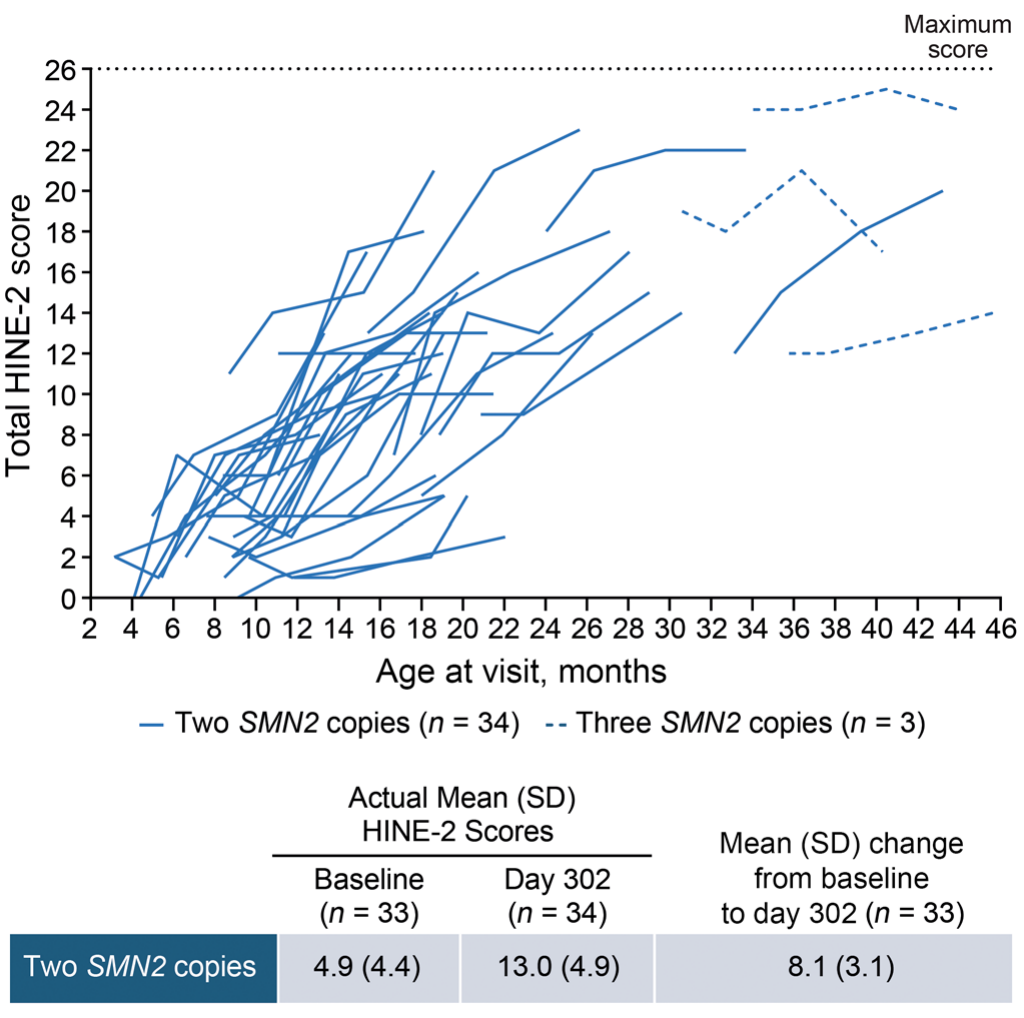
Changes in HINE-2 total score by age at visit. All data from baseline to day 302 are shown for participants with post-baseline scores. Mean scores and change from baseline are not shown for participants with 3 *SMN2* copies due to small sample size.

**Figure 3:**
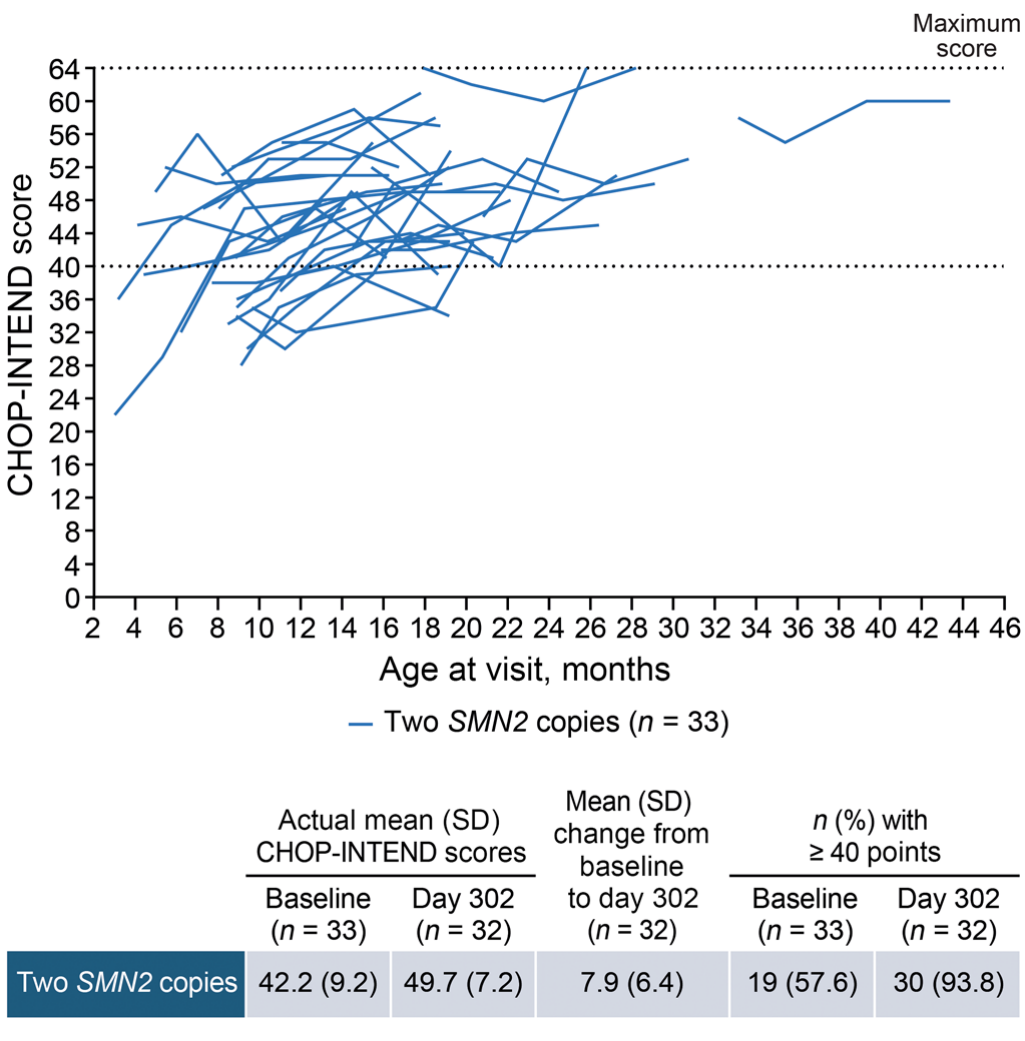
Changes in CHOP-INTEND total score by age at visit. All data from baseline to day 302 are shown for participants with post-baseline scores. CHOP-INTEND was not administered to 4 participants per protocol because they were ≥2 years of age at the time of informed consent and had achieved sitting without support (*n* = 1 in the 2-*SMN2*-copy group and *n* = 3 in the 3-*SMN2*-copy group). An improvement up to or above 40 points in CHOP-INTEND is very uncommon in the natural history of infantile-onset SMA and would indicate a favorable treatment effect (69). CHOP-INTEND, Children’s Hospital of Philadelphia Infant Test of Neuromuscular Disorders.

**Figure 4:**
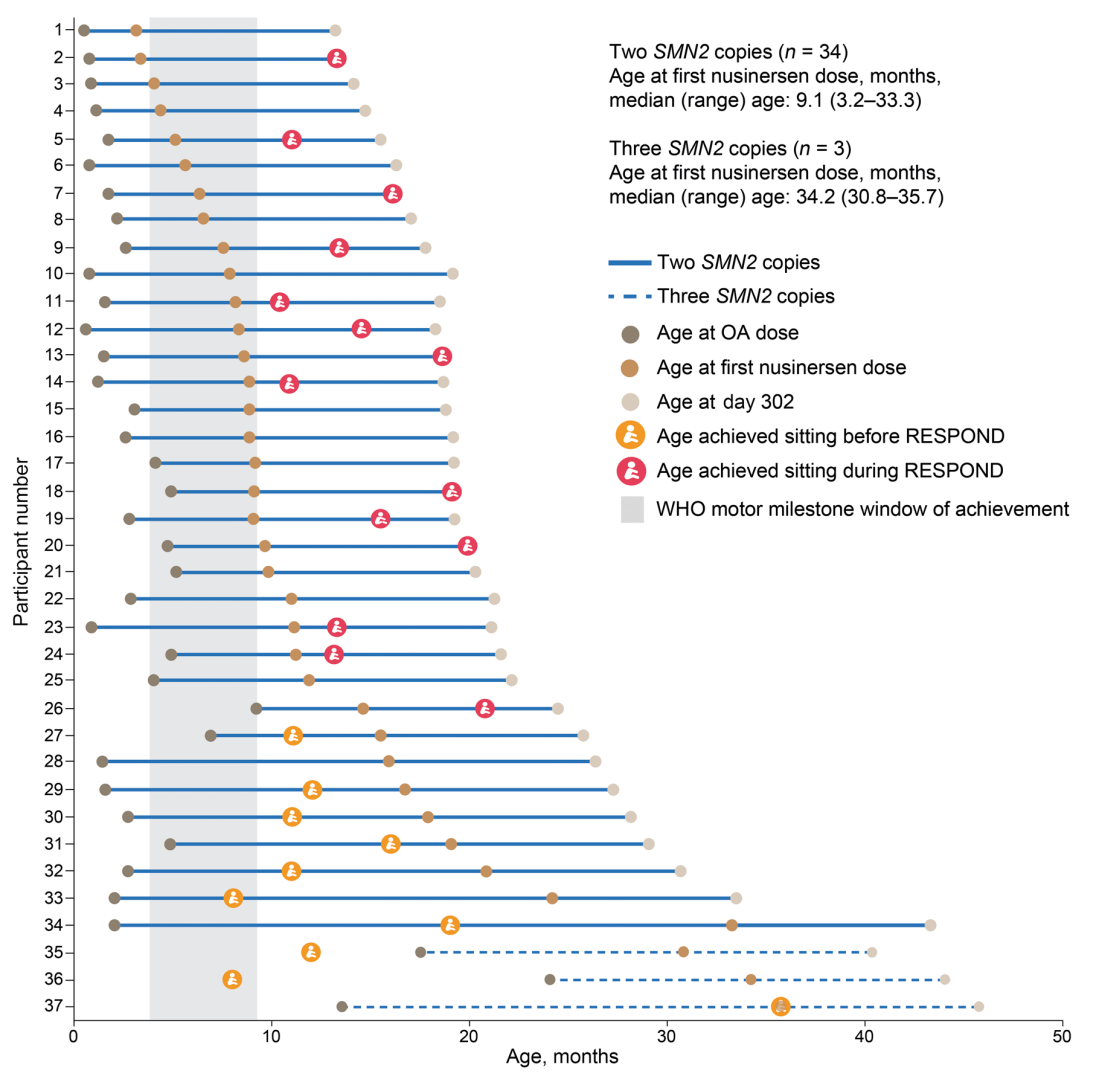
Achievement of independent sitting (WHO motor milestone) on day 302. Shading indicates the WHO motor milestone 1st and 99th percentiles of age at achievement for typical healthy developing children (3.8 and 9.2 months for sitting without support) (24). Only the achievement of sitting without support was examined in the interim analysis; other milestones are planned to be evaluated at the end of the study.

Improvements in motor function and milestones were observed from baseline to day 302. In the 2-*SMN2*-copy group, the mean Hammersmith Infant Neurological Examination section 2 (HINE-2) total score increased by 8.1 points, from 4.9 points at baseline to 13.0 points on day 302 for those with 2 *SMN2* copies (Figure 2). Similarly, the mean Children’s Hospital of Philadelphia Infant Test of Neuromuscular Disorders (CHOP-INTEND) total score increased by 7.9 points, from 42.2 points at baseline to 49.7 points on day 302 in this group (Figure 3); 24 (75%) participants achieved a ≥4-point increase. Of 27 participants unable to sit at baseline, 14 (52%) achieved sitting by day 302, as per the WHO motor milestones assessment (Figure 4).

Improvements were observed across motor function and milestones outcomes for both the younger (aged ≤9 months) and older (aged >9 months) groups with 2 *SMN2* copies (Supplemental Table 3). The mean change in HINE-2 total score from baseline to day 302 was +8.7 points in the younger group and +6.9 points in the older group with 2 *SMN2* copies. The mean change in CHOP-INTEND total scores was +9.3 points and +5.4 points in each group, respectively, with 17 (81%) and 7 (64%) achieving a ≥4-point increase (Supplemental Table 3).

Among the 3 participants with 3 *SMN2* copies, HINE-2 total scores generally remained stable. Since all 3 participants were ≥2 years old and had attained sitting at baseline, CHOP-INTEND was not assessed.

### Changes in biomarkers.

**Figure 5:**
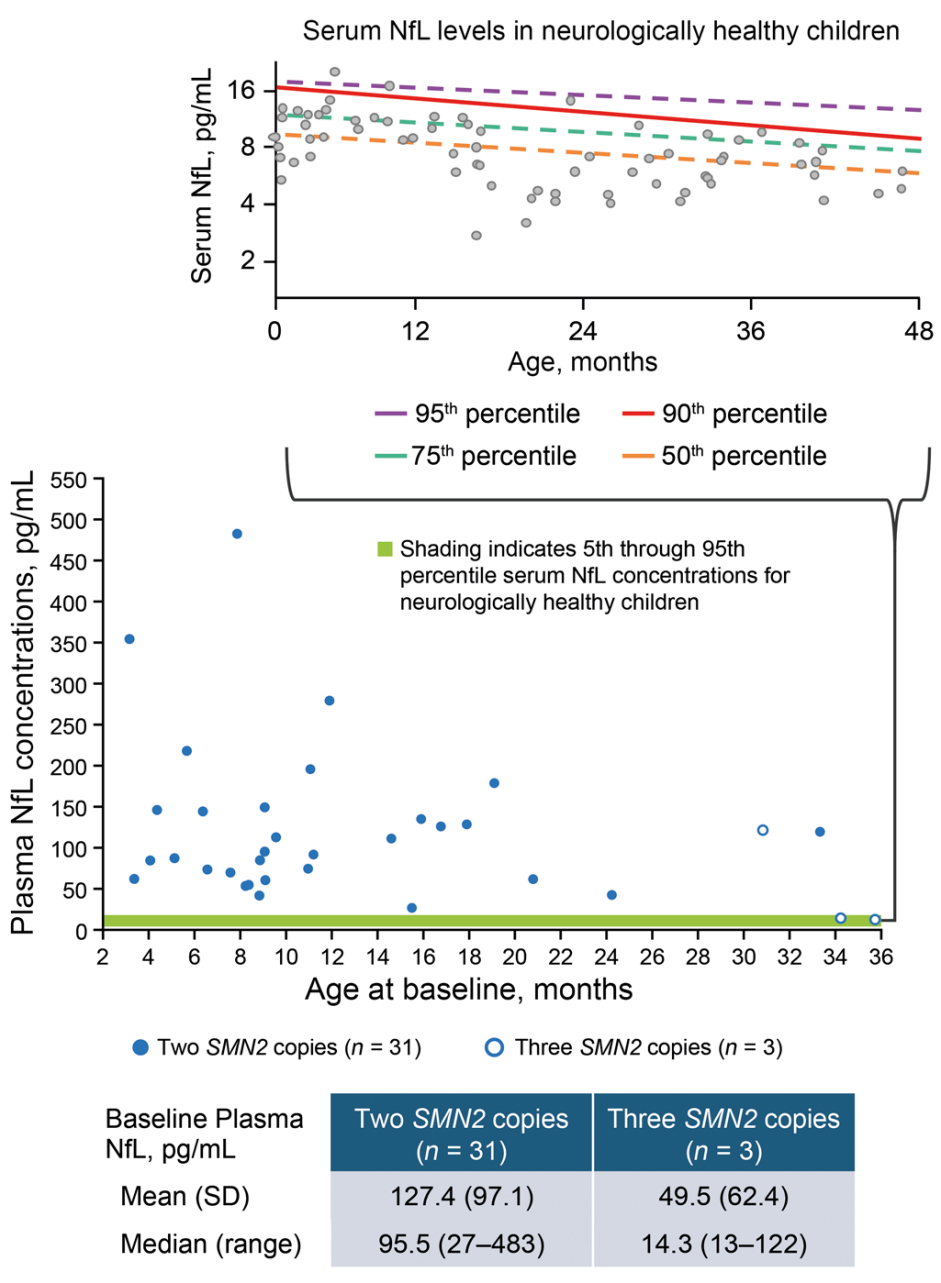
Baseline plasma NfL concentrations by age. The top panel shows serum NfL levels in neurologically healthy children (adapted from ref. 25). The bottom panel shows data from RESPOND participants with non-missing baseline values. Although caution is needed when comparing values across studies due to known variations in analytical methods, as well as differences between serum and plasma levels, the baseline plasma NfL levels observed in the RESPOND study (range: 13–483 pg/mL) are substantially higher than the serum NfL levels previously reported for neurologically healthy children (25, 26), which also used single molecular array (Simoa) immunoassay. NfL levels are expected to be approximately 5%–20% higher in serum as compared with plasma (43–45).

**Figure 6:**
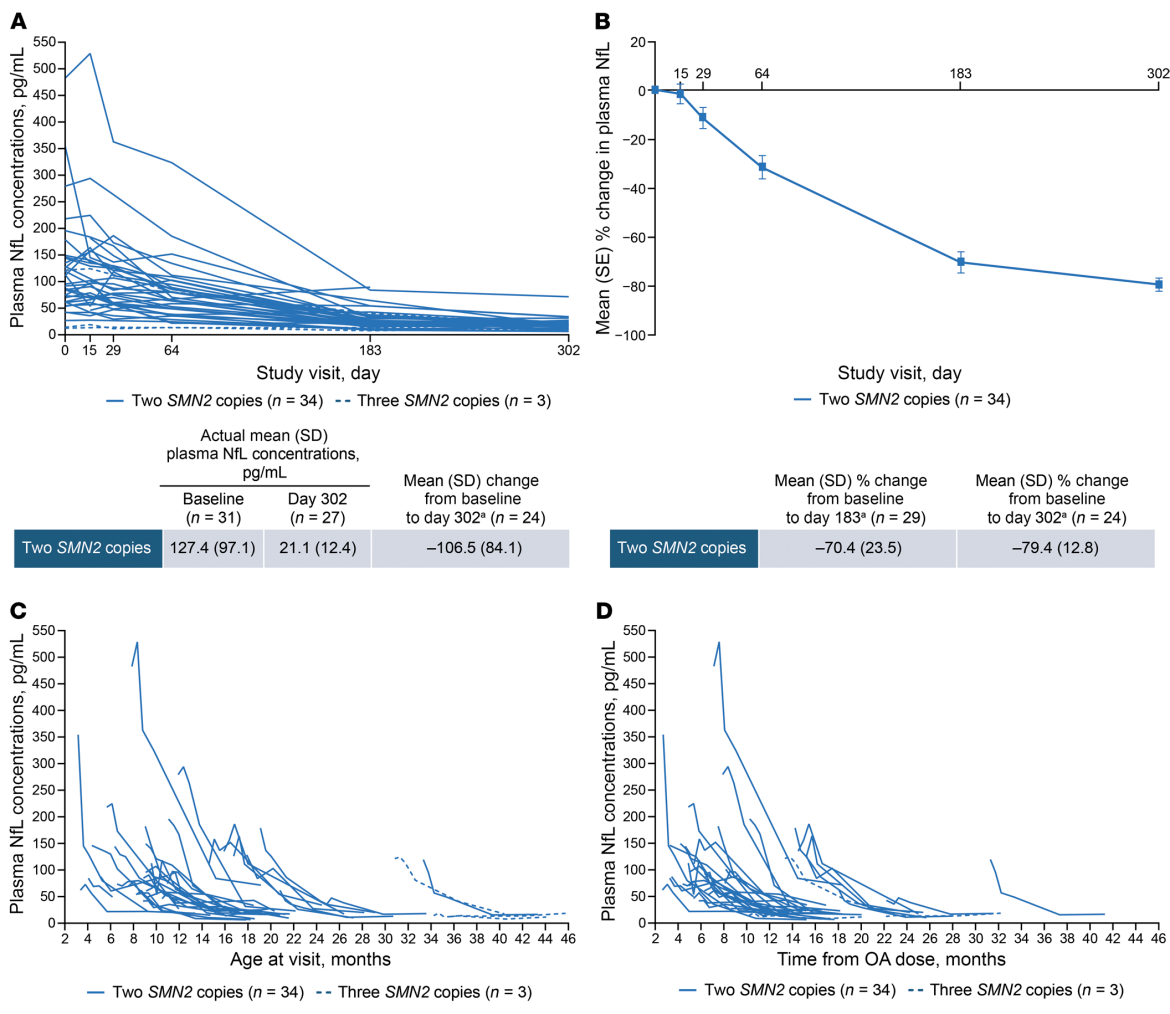
Changes in plasma NfL concentrations. (**A**) Absolute changes by study visit. (**B**) Percent changes by study visit. (**C**) Absolute changes by age at visit. (**D**) Absolute changes by time from OA. All data from baseline to day 302 are shown for participants with postbaseline scores. Individual participant trajectories may overlap. Descriptive statistics are not shown for participants with 3 *SMN2* copies due to small sample size. ^a^Mean (SD) changes were calculated for participants who had assessments at each time point at the time of the NfL data cut.

Baseline plasma neurofilament light chain (NfL) levels were elevated compared with serum NfL levels previously reported in neurologically healthy children of similar ages, indicating active neurodegeneration at study entry (Figure 5) (25, 26). Mean plasma NfL levels rapidly decreased from baseline to day 183 and remained low on day 302, showing a 79% reduction from baseline on day 302 in the 2-*SMN2*-copy group (Figure 6, A and B). Reductions in plasma NfL levels were observed regardless of age at nusinersen initiation or time since OA administration (Figure 6, C and D, and Supplemental Table 3). Similar patterns of reduction were observed in cerebrospinal fluid (CSF) NfL levels, with an 82% reduction during the same period (Supplemental Figure 1). Among participants in the 3-*SMN2*-copy group, 1 participant with an elevated baseline plasma NfL level experienced a reduction similar to that observed in the 2-*SMN2*-copy group. The levels remained low over time for the other 2 participants in this group.

**Figure 7:**
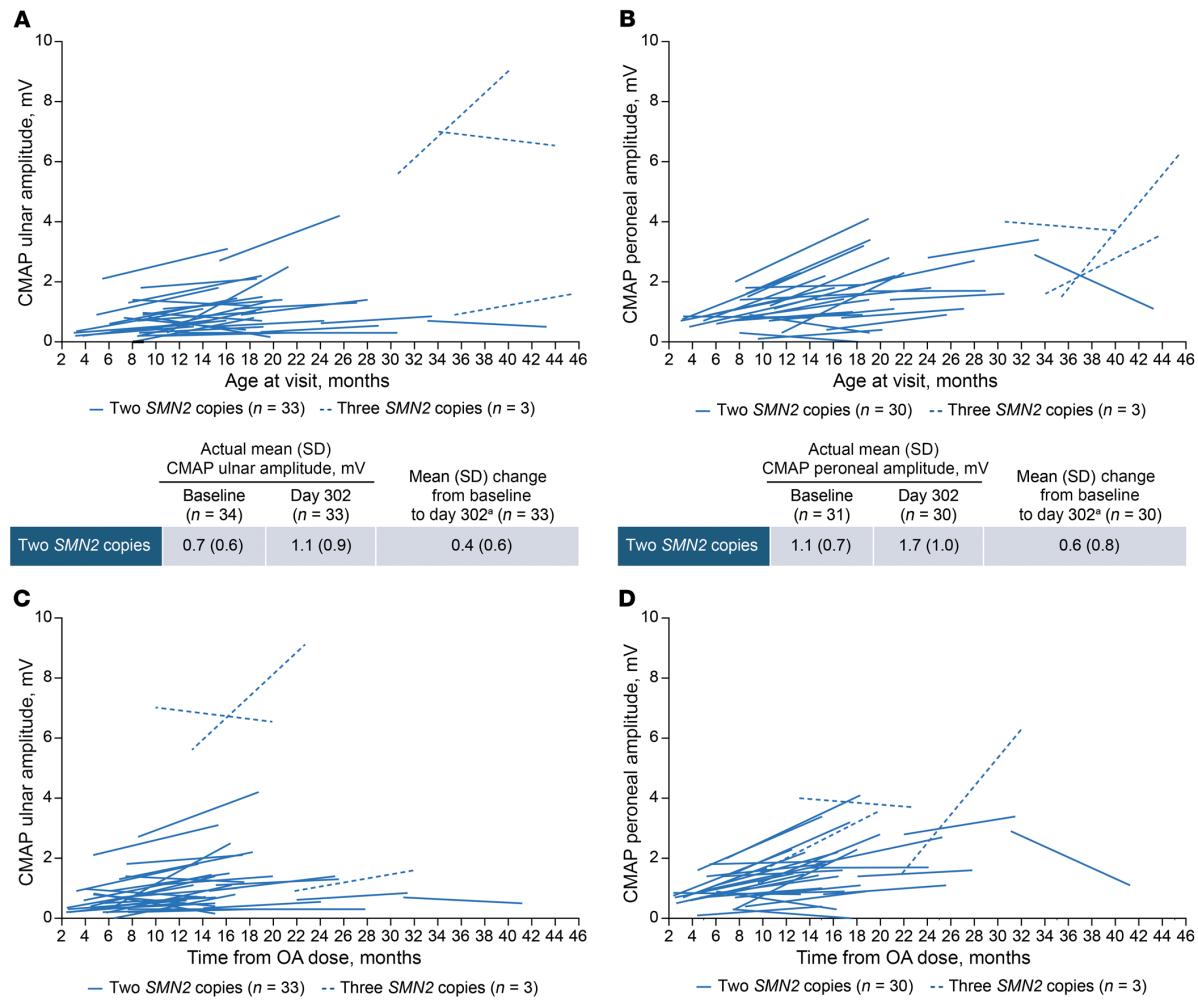
Changes in ulnar and peroneal CMAP amplitude. Changes by age at visit (**A** and **B**) and time from OA (**C** and **D**). All data from baseline to day 302 are shown for participants with postbaseline scores. Descriptive statistics are not shown for participants with 3 *SMN2* copies due to small sample size. ^a^Mean (SD) changes were calculated for participants who had assessments at each time point at the time of the data cut.

Improvements in mean ulnar and peroneal CMAP amplitudes were observed from baseline on day 302, with mean increases of 0.4 mV and 0.6 mV for ulnar and peroneal amplitudes, respectively (Figure 7, A and B) in the 2-*SMN2*-copy group. The pattern of improvement in CMAP was similar regardless of age at nusinersen initiation and time from OA dose (Figure 7, C and D, and Supplemental Table 3). Ulnar and peroneal CMAP amplitudes increased in 2 of the 3 participants in the 3-*SMN2*-copy group, while the participant with the highest baseline value experienced a slight decrease.

### Changes in investigator- and caregiver-reported outcomes.

**Figure 8:**
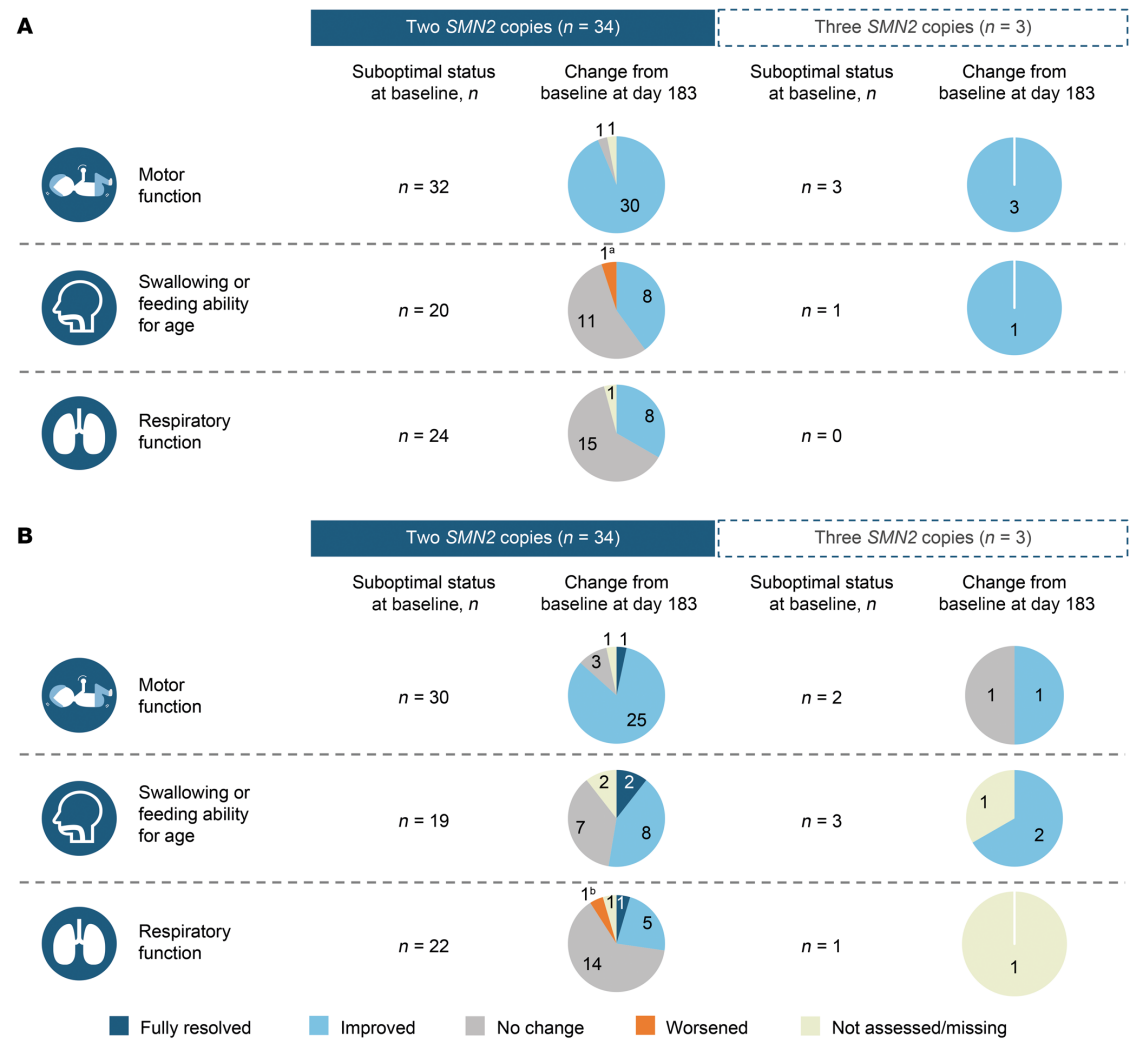
Changes in suboptimal clinical status on day 183. Changes according to (**A**) investigator and (**B**) caregiver assessment. Changes on day 183 are reported for this outcome because the assessment was not performed on day 302 per the protocol. The language used on the form for caregiver was “strength and ability to move (example: unable to sit at the appropriate age),” “ability to breathe (example: requires ventilator support),” “ability to swallow (example: chokes when drinking liquids),” and “other”. In **A**, 4 participants with 2 *SMN2* copies were reported to have suboptimal status in the “other” category at baseline. On day 183, no change was reported in 1 participant, and the 3 other participants were not assessed within this domain on day 183. In **B**, 6 participants with 2 *SMN2* copies and 1 participant with 3 *SMN2* copies were reported to have suboptimal status in the “other” category at baseline. On day 183, 2 participants with 2 *SMN2* copies were reported to have “improved” and the other 4 were “not assessed” within this domain, and the participant with 3 *SMN2* copies was reported to have “improved.” ^a^A gastrointestinal tube was placed in the participant on day 85. ^b^Participant had 2 severe AEs (acute/chronic respiratory failure) <2 months prior to day 183. Both events were unrelated to the study drug and were resolved.

In the 2-*SMN2*-copy group, improvements were reported by day 183 for 30 (94%) participants with investigator-reported suboptimal motor function, 8 (40%) with suboptimal swallowing or feeding ability, and 8 (33%) with suboptimal respiratory function at baseline. No changes were reported by 1 (3%), 11 (55%), and 15 (63%) participants, respectively. In the 3-*SMN2*-copy group, all 3 (100%) participants with suboptimal motor function and 1(100%) with suboptimal swallowing or feeding ability at baseline reported improvement on day 183 (Figure 8A). The majority of caregivers observed improvements in suboptimal motor function and swallowing or feeding ability, while respiratory function remained unchanged in many participants (Figure 8B).

**Figure 9:**
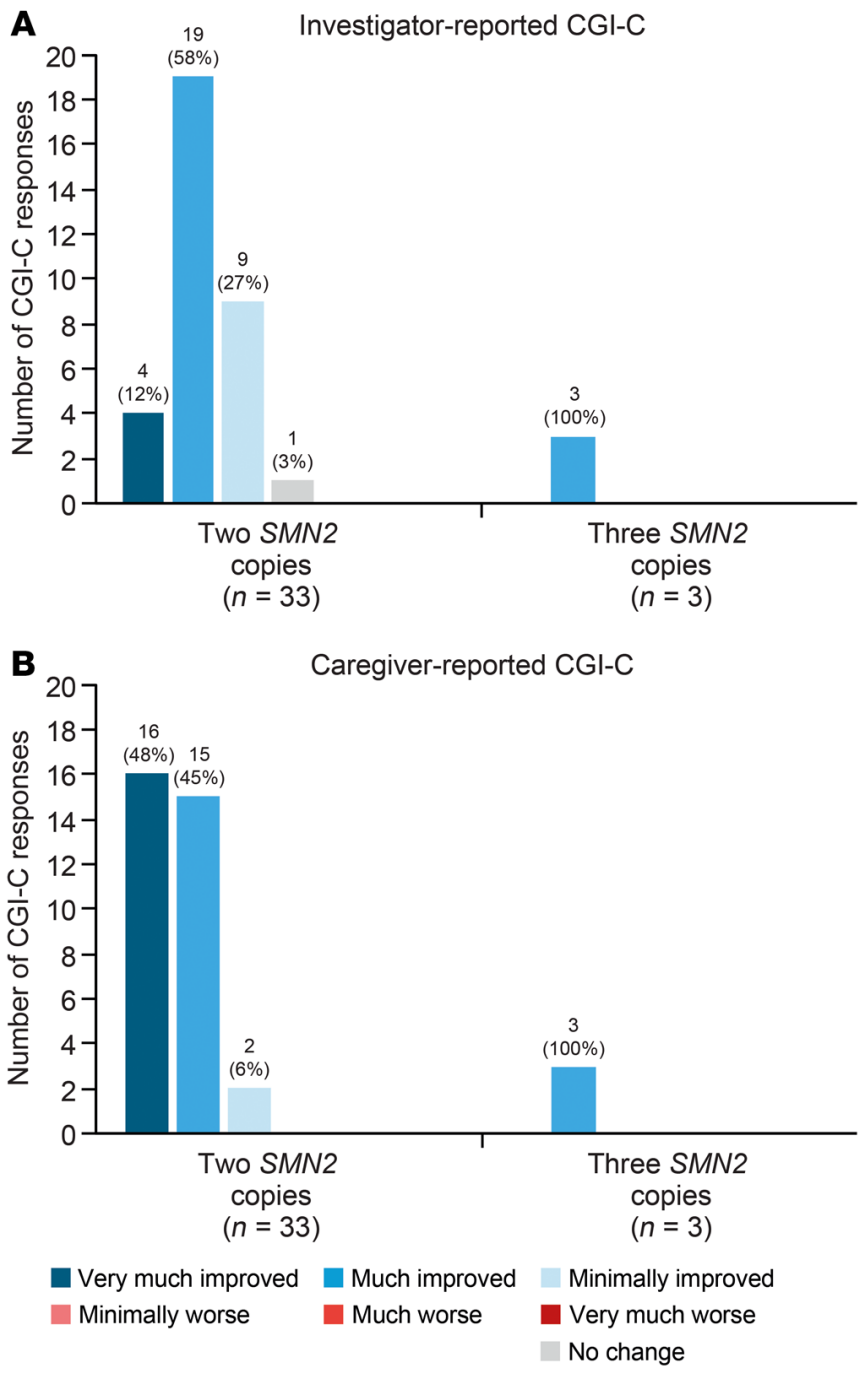
Proportion of CGI-C responders on day 302. Proportion per (**A**) investigator and (**B**) caregiver assessment. CGI-C was assessed by both the investigator and caregiver using a 7-point scale, ranging from “very much improved” to “very much worsened,” to rate overall improvement since study enrollment.

Most investigators and caregivers observed global improvements as assessed by Clinical Global Impression of Change (CGI-C) on day 302, reporting that participants had “very much improved,” “much improved,” or “minimally improved” (Figure 9). Seventy percent of participants with 2 *SMN2* copies and all participants with 3 *SMN2* copies were assessed by the investigators as either “very much improved” or “much improved.” “No change from baseline” was reported by the investigator for 1 participant with 2 *SMN2* copies (3%), and no cases of worsening were reported.

### Safety and tolerability.

The majority of adverse events (AEs) reported were mild to moderate in severity (Table 2). Overall, serious AEs were reported in 17 (37%) participants, and none of these were considered to be related to nusinersen by the investigator. Mild AEs of proteinuria that occurred in 3 (7%) participants were considered to be related to nusinersen by the investigator; all 3 participants continued to receive nusinersen treatment. One death from respiratory arrest was reported, which was not considered to be related to nusinersen by the investigator. The event happened after completion of the last dose of nusinersen in the study but prior to completion of the end of study visit (a nondosing visit).

The most common AEs reported by ≥15% of participants were upper respiratory tract infections, pyrexia, pneumonia, viral upper respiratory infections, and vomiting. No clinically relevant trends related to nusinersen in hematology, blood chemistry, urinalysis, coagulation, vital signs, ECGs, or liver function tests were observed.

## Discussion

Although treatment with OA has improved survival and motor function in children with SMA, preclinical animal models and human postmortem studies suggest that motor neuron transduction remains incomplete, with reported rates varying between 40% and 90% (17–19). The consequences of untransduced motor neurons may not become apparent for years because motor neuron degeneration precedes clinical symptomatology in SMA (27). The RESPOND study is based on the hypothesis that administrating nusinersen could increase SMN protein in untransduced motor neurons, leading to additional clinical benefits for individuals with SMA.

Results from the RESPOND study demonstrate that (i) consistent with the investigator’s assessment of suboptimal clinical status, participants showed active neurodegeneration and severe denervation at study entry despite prior treatment with OA, and (ii) participants experienced improvements in clinical and biomarker outcomes following initiation of nusinersen, with no new safety concerns identified. Our study addresses a critical and timely clinical question regarding the need for additional therapies in individuals with SMA who show suboptimal clinical status after OA. The use of NfL and CMAP as objective biomarkers to support clinical observations enhances biological understanding and suggests a potential approach to identifying patients suitable for early add-on treatment, with the goal of optimizing outcomes.

Growing evidence from real-world cohort studies indicates that many individuals treated with OA have suboptimal clinical status (12, 15, 16, 28–30). Data from larger international registries indicate that motor delays are common in children treated with OA at an older age after symptom onset, but delays are also observed in younger children identified through newborn screening (12, 28). Two recent multicenter US studies similarly reported ongoing disability, motor delays, or suboptimal outcomes in many OA-treated children. Suboptimal outcomes were particularly common (~50%) among children with 2 *SMN2* copies, though they were also seen in some with 3 copies, many of whom required additional *SMN2*-modifying therapy (29, 30).

Most participants in RESPOND exhibited multiple domains of suboptimal clinical status at baseline as assessed by the investigator. Nearly all participants, including those with 3 *SMN2* copies, reported suboptimal motor function. These subjective assessments by investigators were further supported by objective measurements of elevated NfL levels and low CMAPs at baseline. Despite the expected limited potential for improvement given their baseline characteristics, participants demonstrated measurable improvements across various outcomes during the study. At the time of study design, greater improvements were expected in younger participants who received nusinersen as early as possible in their disease course. Prior studies suggest that treatment may be most effective before significant motor neuron loss occurs, while enough neurons remain to respond to SMN protein restoration (30). Nevertheless, the study enrolled participants with a wide range of ages at first nusinersen dose (2–36 months), given the lack of consensus on the optimal therapeutic window. Age subgroup analyses used a predefined cutoff of 9 months at first nusinersen dose, based on the typical developmental window for detecting delays in sitting without support. Although mean motor function score changes were slightly greater in the younger group than in the older group, improvements in clinical and biomarker outcomes were observed in both age groups, suggesting that the older group with a longer time since OA also experienced improvements. Therefore, the main analyses combined all participants with 2 *SMN2* copies.

On day 302, nearly all participants (35 [95%]) demonstrated improvements in motor function as measured by HINE-2 total motor milestone scores or CHOP-INTEND total scores. Two participants with 3 *SMN2* copies, who had the highest HINE-2 total scores at baseline, did not report numerical improvements on day 302. However, their investigators reported improvements in their suboptimal motor function on day 183, and both investigators and caregivers reported improvements in CGI-C on day 302. To our knowledge, no threshold has been established to define a meaningful response in HINE-2 total scores. However, since HINE-2 total scores reflect the achievement of key developmental milestones, any improvement may be considered meaningful for these children and their caregivers (31).

Due to differences in study design, participant characteristics, and prior treatment status, the HINE-2 and CHOP-INTEND total score results from this study cannot be directly compared with those from other trials of nusinersen or OA. For example, symptomatic, treatment-naive participants in the ENDEAR trial had a severe disease burden, with low mean baseline HINE-2 and CHOP-INTEND scores (1.3 and 27.9 points, respectively) (32). Nevertheless, among treated participants with 2 *SMN2* copies, mean improvements in HINE-2 total score were greater in RESPOND than in ENDEAR (+8.1 vs. +4.6 points on day 302), suggesting greater motor milestone gains in RESPOND. In contrast, mean improvements in CHOP-INTEND, a motor skill assessment more suitable for weaker infants and younger children (33), were greater in ENDEAR (+11.3 vs. +7.9 points) (32). In the STR1VE and STR1VE-EU studies of symptomatic participants with 2 *SMN2* copies treated with OA, the greatest CHOP-INTEND improvements were seen within the first 3–6 months of OA treatment, after which gains slowed (13, 14). As only a few participants in STR1VE and STR1VE-EU had CHOP-INTEND data beyond 12 months, and HINE-2 data were not collected in these studies, further contextualization with our study is limited. Many RESPOND participants enrolled several months after receiving OA (median time from OA: 6.3 months in participants with 2 *SMN2* copies).

Although age at baseline largely overlapped with or exceeded the typical window for the sitting achievement in healthy children (3.8–9.2 months) (24), most participants (27 of 37 [73%]) were unable to sit independently at baseline. Of the 10 participants who could sit, only 2 achieved this milestone within the normal developmental window. These findings highlight the ongoing unmet need in this population, despite receipt of treatment with OA. Among the participants who could not sit independently at baseline, 14 of 27 (52%) gained the ability to do so by day 302, representing a clinically relevant benefit for these individuals.

None of the 3 participants with 3 *SMN2* copies (age range: 30.8–35.7 months) were able to walk without support at baseline, despite their age exceeding the typical window for walking achievement in healthy children (8.2–17.6 months) (24). Achievements in these key motor milestones will continue to be evaluated during the study.

In addition to measurable motor function improvements, biomarker data supported the additional benefit of nusinersen for patients previously treated with OA. Our findings highlight the potential of neurofilament levels to serve as a key biomarker for assessing disease activity and monitoring treatment response in SMA. Neurofilaments are structural proteins that are released into the interstitial fluid following axonal damage or neuronal degeneration (34, 35), providing quantitative and real-time information on the extent of ongoing neuroaxonal injury (36).

Neurofilaments have been studied extensively as a biomarker across a wide range of neurodegenerative diseases (36). In SMA, neurofilament levels are elevated — with the highest levels observed in the youngest patients with the more severe forms of the disease (37–40). Similarly, in amyotrophic lateral sclerosis (ALS), levels of neurofilament are prognostic for disease progression and survival (41). In the case of *SOD1*-ALS, treatment-driven reductions preceded and predicted clinical benefit over time (42).

In the RESPOND study, most participants exhibited elevated NfL levels at baseline compared with the levels reported in neurologically healthy children (25, 26), suggesting active neurodegeneration at study entry, consistent with the study hypothesis. In a study estimating age-specific reference levels for serum NfL in neurologically healthy children, the following 5% and 95% percentile levels were reported: 5.0 and 18.2 pg/mL for ages 0–1 year, 4.5 and 16.6 pg/mL for ages 1–2 years, and 4.1 and 15.0 pg/mL for ages 2–4 years (25). Similar serum NfL levels were observed in another study that reported 99th percentiles of 22, 20.4, and 18.9 pg/mL for those aged 1, 2, and 3 years, respectively (26). Higher levels in younger infants and children are likely due to high cell turnover during neuronal migration and differentiation in the developing brain (25, 37, 39).

Although caution is needed when comparing values across studies due to known variations in analytical methods, as well as differences between serum and plasma levels, baseline plasma NfL levels observed in the RESPOND study (median [range]: 93.6 [13–483] pg/mL) were substantially higher than serum NfL levels previously reported for neurologically healthy children in these studies (25, 26), which also used single molecular array (Simoa) immunoassay. As NfL levels are expected to be approximately 5%–20% higher in serum as compared with plasma (43–45), our findings of relative elevation of NfL in plasma are likely conservative.

Nearly all RESPOND participants experienced a rapid decline in NfL levels during the loading phase of nusinersen, followed by relative stabilization thereafter — a pattern consistently observed in other nusinersen studies (37, 38, 46). This pattern was observed regardless of age at baseline or time from OA, suggesting that these reductions were not fully driven by age-related declines in NfL levels or transient increases in NfL levels after OA.

Limited data are available on changes in NfL among individuals receiving OA. Evidence suggests that NfL levels increase for up to approximately 6 months after OA administration (47–50). The transient increase in NfL may reflect an inflammatory response in the central nervous system caused by the AAV9 vector or AAV-induced toxicity (47–50).

Approximately half of the participants in the study had more than 6 months between OA and first nusinersen dose (median time from OA: 6.3 months in the 2-*SMN2*-copy group and 13.3 months in the 3-*SMN2*-copy group). Given that these participants had passed the period of transient neurofilament increases after OA, the reductions observed following nusinersen initiation can be largely attributed to nusinersen, rather than resolution of the OA-related elevations.

Consistent with elevations in plasma NfL, RESPOND participants had low CMAP values at baseline, indicating severe denervation at study entry. Median values of ulnar and peroneal amplitudes were 0.6 mV and 0.9 mV at baseline for those with 2 *SMN2* copies, with 79% and 58% of participants showing amplitudes below 1 mV, respectively. In children without neurological disease, mean ulnar and peroneal CMAP values increase from 3.11 mV and 2.68 mV at 1–6 months of age, respectively, to 4.55 mV and 3.69 mV at 12–24 months of age (51). In the natural history of untreated infantile-onset SMA, CMAP amplitudes decline over time, with low values (<1.0 mV) reported for nearly all individuals (52). The threshold of CMAP amplitude used in prior clinical trials enrolling presymptomatic infants with SMA ranged between 1 and 2 mV (10).

In prior studies of nusinersen or OA, increases in CMAP have been generally observed following treatments (10, 53–55). In the START and STR1VE-US studies, symptomatic participants with 2 *SMN2* copies treated with intravenous OA showed mean peroneal CMAP amplitude increases from baseline (mean age: 3.6 months) up to 18 months of age. However, wide variability in CMAP values was observed during follow-up (54). In another study that examined the potential utility of CMAP in predicting motor recovery after OA, individuals with baseline CMAP values <0.5 mV were less likely to achieve independent sitting after OA (53).

Although there is no consensus on defining the threshold for low CMAP values or a clinically meaningful increased response, the low baseline values observed in the RESPOND study likely indicate suboptimal response to OA and a risk of limited potential improvements without additional intervention. Despite the extent of denervation at baseline, improvements in CMAP were observed regardless of age and time from OA, indicating electrophysiologic response to treatment with additional nusinersen, coinciding with motor function gains in other measures.

Improvements were also consistently observed for investigator- and caregiver-reported outcomes. Most participants with suboptimal motor function at baseline experienced improvements following initiation of nusinersen, as assessed by both investigators and caregivers. Caregivers reported improvements in suboptimal swallowing/feeding ability more frequently than investigators. As caregivers are primarily responsible for providing meals to their child, they may be more perceptive of benefits in this domain. Suboptimal respiratory function remained stable for most participants. The RESPOND trial will continue to evaluate these changes through the end of the study.

Given the limited data on the safety of additional therapies and the potential for new safety concerns in individuals receiving nusinersen after OA treatment, evaluating safety was an important objective of this study. Nusinersen has generally been well tolerated in clinical trials and postmarketing safety studies, with most reported AEs and serious AEs (SAEs) consistent with those typically observed in individuals with SMA or associated with lumbar puncture procedures (56). Key safety concerns regarding OA treatment include risk of liver toxicity and thrombocytopenia (13, 14). Monitoring of liver function and coagulation parameters including platelets was performed throughout the study. AEs and SAEs in this study have generally been consistent with prior experience with nusinersen and with reported data from OA trials, with no new safety concerns identified. Given the increasing use of combination or additional therapies and the underlying pathology in this patient population, in which impaired function of peripheral organs may occur (57), future research on multiple SMA treatments should continue to carefully evaluate safety.

There are limited studies examining additional treatment after OA in clinical trials and postmarketing settings (15, 58–62). Most studies were single-center and observational, with small sample sizes. In a recent observational study of 23 presymptomatic children, the authors concluded that preemptive dual therapy with either risdiplam or nusinersen after OA was well tolerated but provided limited or no benefit compared with OA monotherapy (15). The study included presymptomatic children with mostly normal muscle ultrasound at baseline. Most children in the dual-therapy group received risdiplam shortly after OA (*n* = 6); only one received nusinersen for a limited time (15). RESPOND participants may be more comparable to the subgroup of 3 children who initially received OA monotherapy but later received nusinersen due to motor delays or residual weakness. However, limited outcomes were reported for this small group. A recent multicenter, retrospective case series reported on 20 children who received risdiplam following OA due to suboptimal clinical status (62). Compared with our study, the children in this case series had a longer average time from OA administration (15.2 months) and were older at the time of additional therapy initiation (mean age: 24.9 months). While the authors reported improvements in some children after risdiplam treatment, the findings based on electronic health records were limited by variability in data collection and outcome reporting. No biomarker data were available to help understand the underlying pathology (62). Differences in study populations and treatments preclude direct comparisons of outcomes between these studies. However, both the observational studies and RESPOND show the considerable unmet clinical need after OA, especially for those with 2 *SMN2* copies. Most participants with 2 *SMN2* copies in the observational study experienced widespread degenerative changes on muscle ultrasound during follow-up (15).

As none of the currently available DMTs result in a cure for SMA, combination and sequential therapies are increasingly being used in practice to optimize clinical outcomes (63, 64). However, assessing the impact of such approaches is challenging due to several factors, including the lack of randomized controlled trials and clinical variability. Our study focused specifically on the outcomes of individuals treated with nusinersen following OA. Other therapeutic combinations or sequences were considered outside the scope of this study. While risdiplam is another SMN2-targeting therapy that may also be used after OA, its mechanism of action and safety profile differ from those of nusinersen. Therefore, these therapies should not be considered to be interchangeable in terms of efficacy or safety.

Our study has several limitations. First, since RESPOND is an open-label trial without a comparator group, not all observed improvements can be directly attributed to nusinersen treatment, as all participants had received OA prior to enrollment, which likely provides ongoing production of SMN protein in those motor neurons that are transduced. Placebo effects or expectation bias from evaluators and caregivers may also have influenced the reported improvements in investigator- and caregiver-assessed outcomes. The study was not designed as a comparative study because recruiting participants to undergo a sham procedure would not have been feasible. The study was not statistically powered to detect significant differences. Second, at the time of study design, there was no clear consensus on what constitutes suboptimal clinical status after OA, particularly given the variability in participant age and clinical characteristics. Accordingly, the definition was based on investigators’ assessments, which could be subjective. Third, details of symptoms at the time of OA could not be examined, as participants received OA months to years prior to study enrollment. Fourth, there is no consensus on clinically meaningful thresholds for many clinical and biomarker outcomes in this study, especially among individuals previously treated with other DMTs. To help contextualize the findings, we provided reference values based on those typically observed in neurologically healthy children and/or untreated individuals with SMA. Fifth, given the wide variability in participant characteristics, the limited sample size to account for this variability, and the near-universal improvements in both biomarker and clinical outcomes, correlation or predictive analyses between these outcomes could not be conducted.

Despite the study’s limitations, objectively and quantitatively assessed biomarkers can enhance the understanding of real-time disease activity, contextualize the clinical outcomes, and complement the findings from a single-arm trial when a randomized trial is not feasible. As clinicians navigate decisions on sequencing or combining treatments for SMA in the absence of clinical trial data, our findings can support informed decision making to optimize outcomes.

In summary, improvements in clinical and biomarker outcomes support the benefit of nusinersen treatment in infants and children with suboptimal clinical status following OA.

## Methods

### Sex as a biological variable

Sex was not considered as a biological variable.

### Study design

RESPOND is a phase IV, multicenter, single-arm, open-label study evaluating nusinersen in infants and children with SMA who have previously received OA and have suboptimal clinical status as determined by the investigator (Figure 1).

### Participants

Participants were included if they (i) had genetic documentation of 5q SMA, (ii) were aged ≤36 months at first nusinersen dose, (iii) had an *SMN2* copy number of ≥1, (iv) received OA ≥2 months prior to the first nusinersen dose, (v) had received intravenous OA per the approved label or local/regional regulations including the steroid regimen and monitoring, (vi) had suboptimal clinical status as determined by the investigator in ≥1 of the following domains: motor function, swallowing or feeding ability for age, respiratory function, or other (Supplemental Table 2), and (vii) had alanine aminotransferase (ALT) and aspartate aminotransferase (AST) levels ≤2 times the upper limit of normal at screening and within 7 days prior to dosing. Those with severe or serious AEs related to OA treatment that were ongoing during screening were excluded. Investigators were asked to provide a detailed description of each participant’s suboptimal clinical status at baseline. Participants received intrathecal nusinersen per the current label (7): 12 mg administered as four loading doses on days 1, 15, 29, and 64, followed by a maintenance dose every 4 months until day 659. Participants had a post-treatment follow-up visit on day 778.

For clinical and biomarker outcomes, participants who received ≥1 dose of nusinersen and had the opportunity to complete the day 302 assessment at the time of data-cut were included (efficacy set). For safety outcomes, all participants who received ≥1 dose of nusinersen were included (safety set). Data cut dates were June 26, 2023, for neurofilament outcomes and October 18, 2023 for all other outcomes.

The full list of investigators, study site personnel, and study sites is provided in Supplemental Methods.

### Outcomes

#### Motor function and milestones.

HINE-2 total motor milestone score was assessed as the primary endpoint. HINE-2 total score comprises the following eight motor milestone categories: voluntary grasp, ability to kick in supine position, head control, rolling, sitting, crawling, standing, and walking. Total score ranges from 0 to 26, with a higher score indicating better motor function (65).

CHOP-INTEND motor function scale was assessed in participants who were <2 years of age or those who were 2 to ≤3 years of age if they had not achieved independent sitting prior to screening. CHOP-INTEND total scores range from 0 to 64, with higher scores indicating better motor function (33). The achievement of independent sitting during the study was assessed using the WHO motor milestone criteria (24).

#### Biomarkers.

Levels of NfL, a marker of axonal injury and neurodegeneration, were examined as a measure of disease activity and treatment response (37–40). Plasma and CSF NfL levels were measured with a single molecular array (Simoa) immunoassay via the HD-X Analyzer (Quanterix). Measurements were performed using the NfL v2 Advantage kit from Quanterix (product 104073; lot 503808).

CMAP, an electrophysiological biomarker, was assessed to obtain physiologic information about motor units (52, 66). CMAP measures the motor response to supramaximal electrical stimulation of a peripheral nerve, with lower CMAP amplitudes reflecting a reduced number of motor axons comprising the motor unit (67). CMAP amplitude was assessed for the ulnar nerve recorded at the abductor digiti minimi muscle, and the peroneal/fibular nerve recorded at the anterior tibialis muscles.

#### Investigator- and caregiver-reported outcomes.

Investigators and caregivers assessed suboptimal clinical status at baseline based on the following domains: motor function (e.g., motor delays, limited spontaneous or antigravity movement, hypotonia), swallowing or feeding ability for participant age (e.g., fatigue during feeding, weakness in sucking/swallowing, the need for a nasogastric tube), respiratory function (e.g., paradoxical breathing, chest deformity, need for non-invasive ventilation, increased respiratory rate, inadequate cough), or any other area of suboptimal clinical status. Changes in the identified domains relative to participants’ status on day 1 were evaluated at later time points.

CGI-C was assessed by both the investigator and caregiver using a 7-point scale, ranging from “very much improved” to “very much worsened,” to rate overall improvement since study enrollment.

#### Bulbar function assessments.

The ability to tolerate swallowing thin liquids was assessed using a video fluoroscopic swallow study (VFSS). The VFSS is a radiographic procedure that provides a direct, dynamic view of oral, pharyngeal, and upper esophageal function during swallowing (68). The most severe form of bolus airway entry observed during the VFSS exam was documented.

#### Safety and tolerability.

Safety and tolerability were assessed by examining the incidence of AEs overall, by severity, and by relationship to nusinersen. Other safety assessments included clinical laboratory parameters, ECGs, and vital signs.

### Statistics

Analyses were stratified by *SMN2* copy number, a strong prognostic modifier of the disease (67), to assess clinical and biomarker outcomes. Additional subgroup analyses by age at first nusinersen dose were conducted using a prespecified cutoff (≤9 months, >9 months at first nusinersen dose) in the 2-*SMN2*-copy group to examine potential differences in outcomes by age. Outcomes were summarized using descriptive statistics. Mean changes from baseline on day 302 were obtained for each outcome, except in individuals with 3 *SMN2* copies due to the small sample size (*n* = 3). Individual trajectories for all participants are shown in the figures where possible.

Per the protocol, the investigator and caregiver assessments of suboptimal clinical status were performed on days 1, 183, and 540, and at the end of the study, but not on day 302. Accordingly, changes on day 183 are summarized for this outcome in this interim analysis. The VFSS was performed on day 1 and at the end of the study, so only baseline data are presented.

### Study approval

RESPOND was conducted in accordance with the approved protocol, the principles of applicable International Council for Harmonisation and Good Clinical Practice guidelines, and the requirements of Clinical Trials Regulation (EU) No. 536/2014 or, as applicable, Clinical Trials Directive 2001/20/EC. The study adheres to the ethical principles outlined in the Declaration of Helsinki, and signed informed consent was collected from the parent or guardian of all participants. Investigators obtained ethics committee approval for the protocol, informed consent forms, and other required study documents prior to starting the study. See Supplemental Methods for RESPOND Ethics Committee approval information.

### Data availability

The materials and data supporting this manuscript are available by request through the Vivli platform (https://vivli.org/) using the search term NCT04488133.

## Author contributions

CMP, RSF, JAP, RM, JFB, NLK, JS, RF, WL, RL, SF, BY, and ADP contributed to the concept and study design, analyzed and interpreted the data, and reviewed and revised the manuscript for intellectual content.

## Funding support

- Biogen.

## Supplementary Material

Supplemental data

ICMJE disclosure forms

## Figures and Tables

**Table 1:**
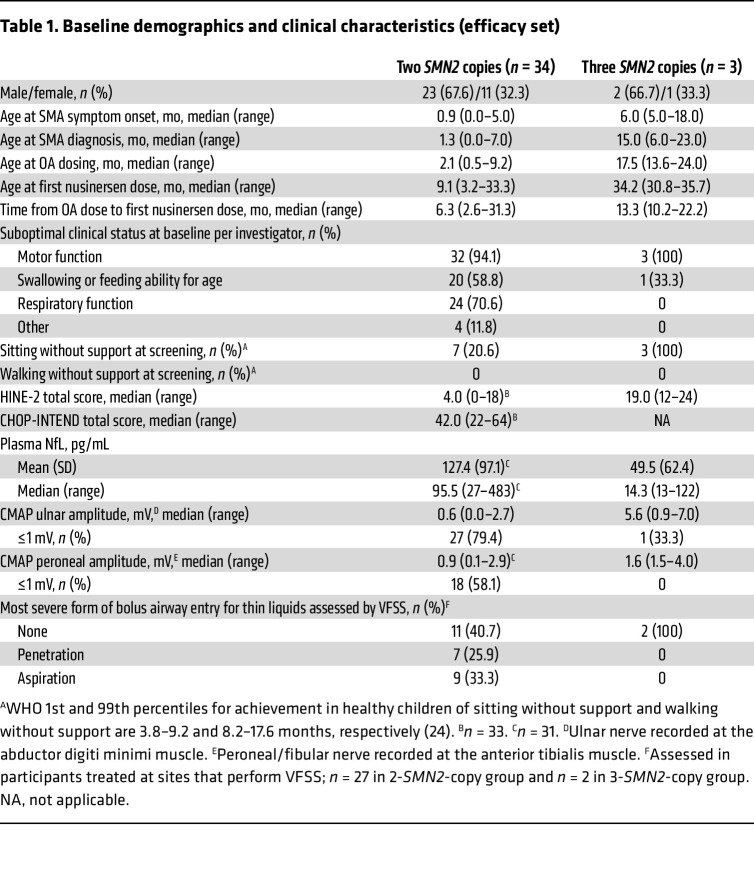
Baseline demographics and clinical characteristics (efficacy set)

**Table 2:**
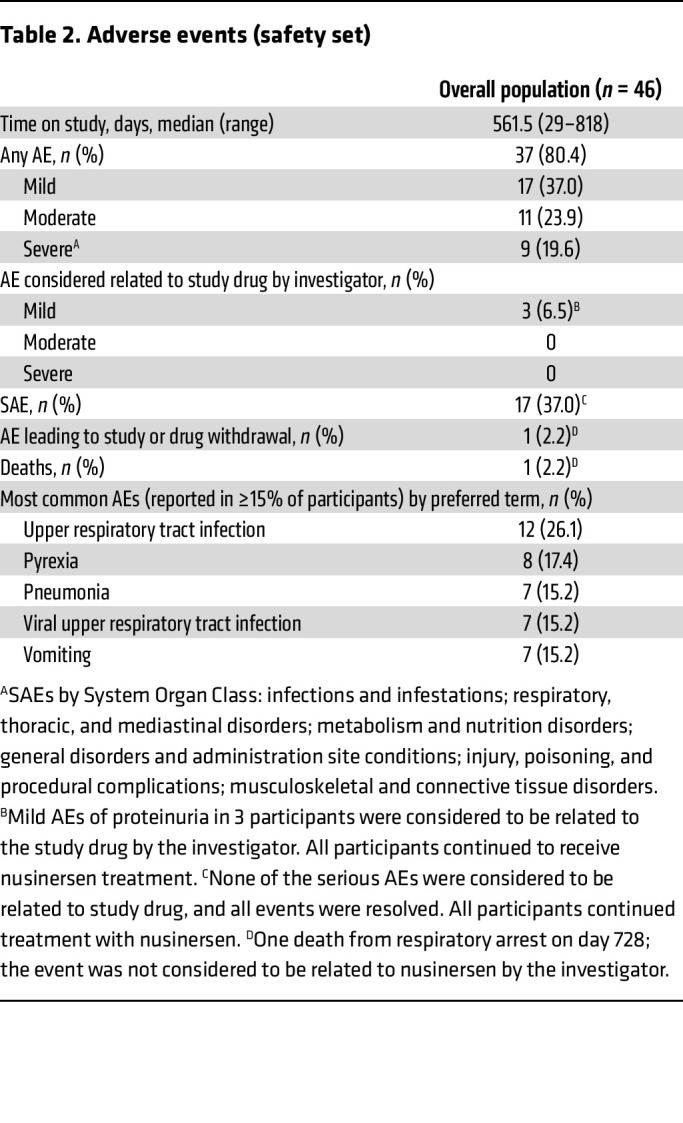
Adverse events (safety set)

## Version 1 09/16/2025

In-Press Preview

## Version 2 11/17/2025

Electronic publication
