# Supplementary material for: Open-label phase IV trial evaluating nusinersen after onasemnogene abeparvovec in children with spinal muscular atrophy
Source: J Clin Invest. 2025 Sep 16;135(22):e193956. doi: 10.1172/JCI193956 (PMC12618081; doi:10.1172/JCI193956)
Supplement: Supplemental data [file jci-135-193956-s267.pdf]

Supplementary Methods

RESPOND Principal Investigators and Site Study Personnel

Principal Investigators and site study personnel at the sites that enrolled:

Germany: Anna Krumbiegel MD, Deike Weiss MD, **Jessika Johannsen MD**, Jonas Denecke MD, Nicole Muschol MD, Paula Steffens MD, Jila Taherpour, Angela Goerke, Kerstin Hallmann, Kerstin Leske, Ines Battermann, Ulrike Petersen, Finja Keinke, Anna-Lena Krumbiegel, Jana Essers, Mathias Gelderblom MD, Ulrike Meyer, Gamze Lika Palabiyik, Frida Tomala.

Israel: Alla Kuzminsky MD, Lital Cohen MD, Shany Lando Dotan MD, **Sharon Aharoni MD**, Tami Schnayderman, Tanya Moss, Yoram Nevo MD, Hanna Eizenberg, Yaara Daud, Miriam Vax.

Italy: **Eugenio Mercuri MD**, Georgia Coratti PhD, Laura Antonaci MD PhD, Marco Piastra MD, Marco Luigetti MD PhD, Maria Carmela Pera MD PhD, Marika Pane MD PhD, Orazio Genovese MD PhD, Roberto De Sanctis, Fabiana Bonetto, Valeria Bovetti, Luisa Cassano, Gianpaolo Cicala MD, Angela Maria Cozzolino, Marta Barba, Miriam Cruciani, Elisa De Sanctis, Roberto Digilio, Emilia Laudati, Katia Mercone, Arianna Oliviero, Lucia Pavan, Diletta Rossi, Maria Rosaria Vizzino; Claudia Dosi MD, Maria Teresa Arnoldi, Paola Lanteri MD, **Riccardo Masson MD**, Stefano Parravicini, Riccardo Zanin, Francesca Andreetta, Angela Pasquariello, Elena Mangia, Emilio Ciusani, Alessandro Campari, Gloria Pome, Marta Rivelli, Maria Cambareri, Monica Ongarato, Stefano Parravicini MD, Maria Arnoldi.

Spain: Gloria Lopez Sobrino MD, Sandra Espinosa MD, Miguel Angel Fernandez Garcia MD, Ramon Velazquez Fragua MD PhD, **Maria Garcia Romero MD**, Samuel Ignacio Pascual Pascual MD PhD, Luis Sanchez Rubio Ferrandez, Monserrat Bret MD PhD, Gloria Giraldez Marin, Marta Calvo Aguilar, Marta Monzon Villar, Ana Isabel Sevillano Blanco, Gema Mora Arenal, Cristina Cardenas Sarralde; **Andres Nascimento Osorio MD PhD**, Carlos Ortez Gonzalez MD PhD, Carlos Ruiz Hernandez MD, Daniel Natera de Benito MD, Jessica Maria Exposito Escudero MD, Julita Medina Cantillo MD PhD, Laura Carrera Garcia MD, Obdulia Moya Arcos, Sandra Roca, Laura Sole Heuberger, Alicia Rodriguez Ramirez, Maria Valle Saez, Isabel Miquel Aymar, Arnau Masanes Pujol, Lorena Munera Bueno, Katherine Viejo Banchon, Alba Murciano Cabeza, Miriam Coto Moreno, Catarina Cindea, Francesca Abate.

During the study Dr Maria Garcia Romero MD assumed responsibility of Principal Investigator from Dr. Samuel Ignacio Pascual Pascual.

United States: Carolyn Kelley, **Julie Parsons MD**, Meghan Moore Burk, Michele Yang MD, Susan Apkon MD, Tasneem Alimam, Ellen Burke, Carla Cobos-Hernandez, Obehi Enabulele, Margaret Finlay, Christiane Furlong, Kristen Gonzales, Katie Gordon, Mary Johnson, Hanalei Lintag, Melissa Munzing, Kalie Nakata, Kirsten Petty, Sydney Popovich, Carla Rameri, Christine Stewart; Tate Keough, Stephanie Manberg DO, Melissa McIntyre, Amelia Wilson, **Russell Butterfield MD PhD**, Berrie Child, Whitney Crosgrove, Hannah Harvey, Cameron Hill, Michelle Hillman, Kristie Holbrook, Jacob Majers, Sarah Moldt, Paymon Mirzaakbari, Cody Nesbit, Michelle Ngo, Winter C. Redd, Jeanine Rushforth, Brittney Scharman, Mindy Suttlemyre, Michael Voight, Melissa Webb, Jason Worthen; Abigail Schwaede MD, Katie Hoffman, Laura Brown, **Nancy Kuntz MD**, Vamshi Rao MD, Anna De Sonia, Mary Wright, Sonny Sherwood, Ami Patel, Tara Mongkolpradit, Yoomi Lee, Amber Buehner, Duncan Schulte, Laurey Brown, Denise Rizzo, Ishita Tejani, Molly Gallivan,

Yvette Ondouah Nzutchi, Ariana Pavlopoulos, Alexis Noirot, Patrick Sohn; Carolina Tesi-Rocha MD, Jacinda Sampson MD PhD, Jenna Klotz MD, Karolina Anna Watson PNP-BC, Richard Steven Gee, Sally Dunaway Young, Shellie Josephs MD, Trinh Tina Duong PhD, **John West Day MD PhD,** Rabia Farooquee, Sai Nimmagadda, Susan Thomas, Karolina Watson, Lesly Welsh; Allan Glanzman, Jean Flickinger, **John Brandsema MD**, Sabrina Yum MD, Susan Matesanz MD, Nicole Brownell, Kristin Cao, Shannon Claire, Alicia Hahn, Ting He, Larisa Horvath, Milan Mahesh, Leah Michalski, Karen Monono, Adam Motsney, Jennifer Nedley, Matthew Rib, Brianna Riggio, Kahreen Samoy Balmes, Andrea Marie Solly, Jennifer Wilson; Aravindhan Veerapandiyan MD, Charles James MD, **Kapil Arya MD**, Kevin Wong MD, Melissa Hicks-Wittman, Michael Nelson, Jill Franklin, Jill Hernandez, Gina Hawkins, Steven Giompoletti, Vik V Kuraku, Kathy Hummel, Ashley Bryan; **Crystal Proud MD**, Elise Darling, Sarah Chagnon MD, Megan Baluyot, Jennifer Beachum, Audrey Giering, Melinda Griggs, Christina Hellauer, Marysha Jones, Alyssa McGowan, Yvonne Moody, Erika Paradiso, Kayla Treneff, Ashley Vasko, Elizabeth Walker, Cassandra Todd, Kelcee Widdess; Meganne Leach PPCNP-BC, Wendy Herrick, Kirsten Zilke, Brittany Gurgel, **Erika Finanger MD**, Bryn McCarthy, Carol Tagorda, Beata Dyar, Anthony Jones, Kim Vongsa, Venessa Zendejas, Evan McKenna, Ping Auyeung, Sarah Bissonette, Parvathi Sidigonde.

RESPOND Ethics Committee approvals

Authority/Committee Name	Country
Ethikkommission der Aerztekammer Hamburg	Germany
Rabin MC Ethics Committee	Israel
Comitato Etico dell'Università Cattolica del Sacro Cuore e annesso Policlinico "A. Gemelli"	Italy
Comitato Etico della Fondazione IRCCS Istituto Neurologico Carlo Besta di Milano	Italy
Hospital Universitari i Politecnic La Fe	Spain
Western Institutional Review Board (Central IRB)	USA
University of Utah IRB	USA
Ann & Robert H. Lurie Children’s Hospital of Chicago Institutional Review Board	USA
Nemours Delaware IRB	USA
Stanford University IRB	USA
The Children’s Hospital of Philadelphia IRB	USA
Oregon Health and Science University IRB	USA
University of Minnesota IRB	USA

Supplementary Table 1. Baseline demographics and clinical characteristics

	**Age at first nusinersen dose group**	
	**≤ 9 months[a]**<br>**Two *SMN2* copies n = 21**	**> 9 months[a]**<br>**Two *SMN2* copies n = 13**
Male/female, n (%)	15 (71.4) / 6 (28.6)	8 (61.5) / 5 (38.5)
Age at SMA symptom onset, months, median (range)	0.7 (0.0–3.0)	1.0 (0.0–5.0)
Age at SMA diagnosis, months, median (range)	1.0 (0.0–5.0)	2.1 (0.0–7.0)
Age at OA dosing, months, median (range)	1.7 (0.5–5.1)	2.7 (0.8–9.2)
Age at first nusinersen dose, months, median (range)	8.2 (3.2–9.8)	15.9 (11.0–33.3)
Time from OA dose to first nusinersen dose, months, median (range)	4.9 (2.6–7.8)	14.2 (5.4–31.3)
Suboptimal clinical status at baseline per investigator, n (%)		
Motor function	20 (95.2)	12 (92.3)
Swallowing or feeding ability for age	14 (66.7)	6 (46.2)
Respiratory function	14 (66.7)	10 (76.9)
Other	3 (14.3)	1 (7.7)
Sitting without support at screening, n (%)[b]	0	7 (53.8)
Walking without support at screening, n (%)[b]	0	0
HINE-2 total score, median (range)	2.0 (0–11)	8.0 (1–18)[c]
CHOP-INTEND total score, median (range)	38.0 (22–52)	44.5 (37–64)[c]
Plasma NfL, pg/mL		
Mean (SD)	132.0 (115.3)[d]	121.0 (68.3)
Median (range)	86.2 (42–483)[d]	119.6 (27–279)
CMAP ulnar amplitude, mV,[e] median (range)	0.6 (0.0–2.1)	0.6 (0.2–2.7)
≤ 1 mV, n (%)	17 (81.0)	10 (76.9)
CMAP peroneal amplitude, mV,[f] median (range)	0.8 (0.1–2.0)[d]	1.2 (0.3–2.9)

≤ 1 mV, n (%)	13 (72.2)	5 (38.5)
Most severe form of bolus airway entry for thin liquids assessed by VFSS, n (%)[g]		
None	7 (43.8)	4 (36.4)
Penetration	4 (25.0)	3 (27.3)
Aspiration	5 (31.3)	4 (36.4)

[a]9 months was defined as 300 days of age. [b]WHO 1st and 99th percentiles for achievement in healthy children of sitting without support and walking without support are 3.8–9.2 and 8.2–17.6 months, respectively.(26) [c]n = 12. [d]n = 18. [e]Ulnar nerve recorded at the abductor digiti minimi muscle. [f]Peroneal/fibular nerve recorded at the anterior tibialis muscle. [g]Assessed in participants treated at sites that perform VFSS, n = 16 in ≤ 9 months two *SMN2* copy group and n = 11 in > 9 months two *SMN2* copy group.

CHOP-INTEND = Children's Hospital of Philadelphia Infant Test of Neuromuscular Disorders; CMAP = compound muscle action potential; HINE-2 = Hammersmith Infant Neurological Examination section 2; NA = not applicable; NfL = neurofilament light chain; OA = onasemnogene abeparvovec; SD = standard deviation; SMA = spinal muscular atrophy; *SMN2 = survival motor neuron 2*; VFSS = video fluoroscopic swallow study.

Supplementary Table 2. Examples of Investigator-Provided Descriptions of Baseline Suboptimal Clinical Status

Suboptimal status at Baseline	Investigator-Provided Descriptions of Baseline Suboptimal Clinical Status
Suboptimal motor function	• Limited antigravity lower extremity movement<br>• Forearm pronation right greater than left indicating contracture<br>• Inability to lift head in prone<br>• Torticollis head turns to left with tight left SCM<br>• Minimal/poor spontaneous movements<br>• No head control/head control not yet completed<br>• Poor antigravity movements<br>• Reduction of antigravity movement of upper and lower limbs<br>• Hypotonia<br>• Significant motor delays (motor milestone acquisition)<br>• CHOP-INTEND <50<br>• No reflexes<br>• Reduction of spontaneous movements<br>• Poor mobility<br>• No sitting position<br>• Weakness<br>• Arm weakness<br>• Generalized reduction of muscle strength<br>• Reduction of upper limb movement<br>• Minimal lower limb movements<br>• No head control on pull to sit or in supported sitting<br>• Weak hand grasp<br>• CMAP poor<br>• Poor spine and lower extremity more weak than upper extremity<br>• No weight bearing on legs<br>• Full head lag with traction<br>• Not rolling<br>• Rolls only to side<br>• Reduced lifting in prone position

	• Not sitting independently, with significant proximal weakening<br>• Needs support with sitting, cannot transition to seated position<br>• Frog legged position when supine<br>• Poor motor intensity<br>• Head lag on pull to sit<br>• Significant slip through at shoulders<br>• Not yet standing independently<br>• Mild spinal curvature reflecting truncal weakness<br>• Very poor leg movement<br>• Abnormal NCV, not normalized post Zolgensma<br>• Unable to raise arms above head in sitting<br>• Loses balance in sitting when reaches out or loss of support<br>• No crawling<br>• Stands and weight bears only with significant support<br>• No walking<br>• Patient has not yet attained previously held skills (crawling) which was lost just prior to Zolgensma infusion<br>• No free rising from ground<br>• No climbing of stairs<br>• Sitting - cannot get to sitting independently<br>• Supine - cannot abduct legs to midline<br>• Standing - cannot bear weight<br>• Crawling - cannot assume quadruped
Suboptimal swallowing function	• Likely microaspiration<br>• Bulbar weakness<br>• Tongue fasciculations<br>• Growth deficiency and poor weight gain<br>• Poor sucking/baby gets tired while sucking<br>• Fatigability during the meal<br>• Inability to swallow<br>• Difficulty swallowing<br>• Partial oral feeding<br>• Need for nasogastric tube

	• Fatigue during suctioning<br>• G-tube in place<br>• Difficulty handling secretions<br>• Poor weight gain, has required nasogastric tube feeds<br>• Coughing with feed<br>• Trouble clearing airway when upset<br>• Weakness of swallowing<br>• NPO<br>• Frank aspiration of thin liquids<br>• PEG after gene therapy<br>• Wet vocal quality when crying
Suboptimal respiratory function	• Paradoxical respiratory movement<br>• Diaphragmatic/belly breathing<br>• Chest deformity<br>• Increased respiratory rate/pattern<br>• Tachypnea<br>• Need for prophylactic NIV<br>• Asymmetrical chest wall movements<br>• Use of BiPAP overnight<br>• Very weak, high risk of respiratory insufficiency<br>• Small chest size relative to head size<br>• Diagnostic and abnormal breath<br>• Bell shaped chest<br>• Scoliosis pectus excavatum<br>• Respiratory weakness<br>• Weak cry<br>• Abnormal breath<br>• Weak cough and sneeze<br>• Requires cough assist<br>• Impaired cough<br>• Mild narrowing of upper chest wall<br>• Use of BiPAP<br>• Need of NIV all night and often during the day, often need aspiration

BiPAP = Bilevel Positive Airway Pressure; CHOP-INTEND = Children's Hospital of Philadelphia Infant Test of Neuromuscular Disorders; G-tube = gastrointestinal tube; NCV = nerve conduction velocity; NIV = non-invasive ventilation; NPO = nothing by mouth; PEG = percutaneous endoscopic gastrostomy; SCM = sternocleidomastoid muscle.

Supplementary Table 3. Clinical outcomes stratified by age at first nusinersen dose

	**Age at first nusinersen dose group**	
	**≤ 9 months**<br>**Two *SMN2* copies**<br>**n = 21**	**> 9 months**<br>**Two *SMN2* copies**<br>**n = 13**
HINE-2 total score, mean (SD)		
Baseline	2.9 (2.5)	8.4 (4.8)[a]
Day 302	11.6 (4.3)	15.2 (5.3)
Change from baseline to Day 302	8.7 (3.1)	6.9 (2.7)[a]
CHOP-INTEND total scores, mean (SD)		
Baseline	39.1 (8.4)	47.5 (8.2)[a]
Day 302	48.4 (6.9)	52.2 (7.5)[b]
Change from baseline to Day 302	9.3 (7.1)	5.4 (3.7)[b]
Number (%) with ≥ 4-point increase from baseline to Day 302	17 (80.9)	7 (63.6)[b]
Plasma NfL concentrations, pg/mL, mean (SD)		
Baseline	132.0 (115.3)[c]	121.0 (68.3)
Day 183	31.4 (21.8)[d]	24.3 (12.5)[a]
% change from baseline to Day 183	−66.8 (28.8)[e]	−75.5 (12.5)[a]
Day 302	23.2 (15.7)[f]	18.6 (5.9)[a]
% change from baseline to Day 302	−76.7 (14.7)[a]	−82.2 (10.4)[a]
Absolute change from baseline to Day 302[g]	−102.7 (103.3)[a]	−110.3 (64.0)[a]
CMAP ulnar amplitude, mV,[h] mean (SD)		
Baseline	0.7 (0.6)	0.8 (0.7)
Day 302	1.1 (0.7)	1.2 (1.2)[a]
Change from baseline to Day 302	0.4 (0.5)	0.4 (0.7)[a]
CMAP peroneal amplitude, mV,[i] mean (SD)		
Baseline	0.9 (0.5)[c]	1.3 (0.8)

Day 302	1.7 (1.1)[c]	1.9 (0.8)[a]
Change from baseline to Day 302	0.7 (0.8)[c]	0.5 (0.9)[a]

[a]n = 12. [b]n = 11. [c]n = 18. [d]n = 20. [e]n = 17. [f]n = 15. [g]Mean (SD) changes were calculated for participants who had assessments at each time point (baseline, Day 183 or Day 302) at the time of the NfL data-cut. Among 21 participants in the ≤ 9 months with two *SMN2* copies group, 12 had assessments at all three visits at the time of the NfL data-cut. Mean (SD) changes from baseline to Day 183 and Day 302 were –67% (28.8) and –77% (14.7) in these participants, respectively. [h]Ulnar nerve recorded at the abductor digiti minimi muscle. [i]Peroneal/fibular nerve recorded at the anterior tibialis muscle.
Mean (SD) changes were calculated for participants who had assessments at each time point at the time of data-cut. CHOP-INTEND was not administered to participants who were ≥ 2 years of age at the time of informed consent and had attained sitting without support per protocol.

CMAP = compound muscle action potential; CHOP-INTEND = Children's Hospital of Philadelphia Infant Test of Neuromuscular Disorders; HINE-2 = Hammersmith Infant Neurological Examination section 2; NfL = neurofilament light chain; SD = standard deviation; *SMN2 = survival motor neuron 2.*

Supplementary Figure 1. Changes in cerebrospinal fluid NfL concentrations: (**A**) absolute changes by study visit, (**B**) percent changes by study visit, (**C**) absolute changes by age at visit, (**D**) absolute changes by time from OA

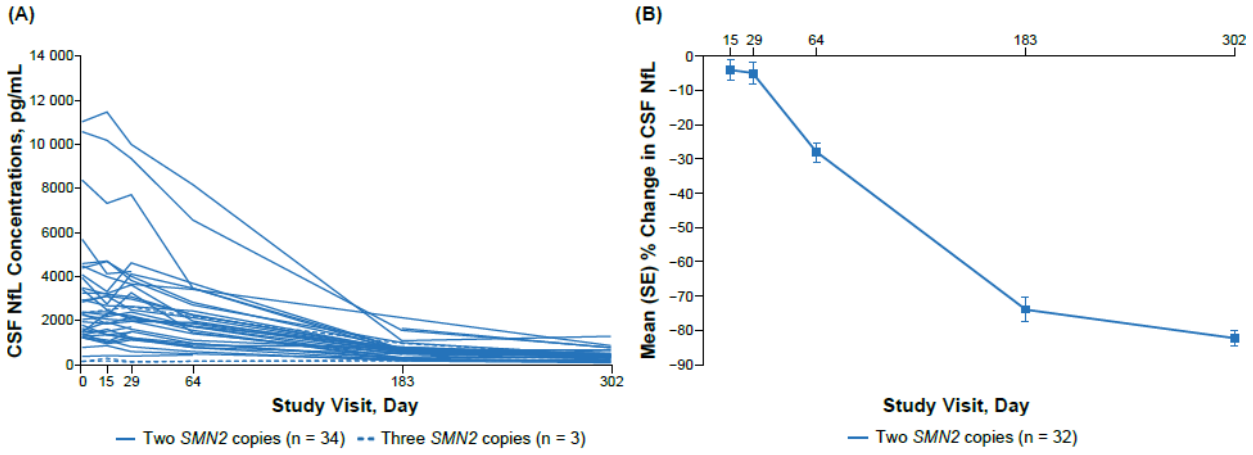

	Actual Mean (SD) CSF NfL concentrations, pg/mL		Mean (SD) Change From Baseline to Day 302[a] (n = 30)
	Baseline (n = 34)	Day 302 (n = 30)	
Two *SMN2* copies	3148.5 (2516.2)	463.6 (257.6)	−2908.4 (2436.7)

	Mean (SD) % Change From Baseline to Day 183[a] (n = 32)	Mean (SD) % Change From Baseline to Day 302[a] (n = 30)
Two *SMN2* copies	−73.9 (19.6)	−82.3 (10.9)

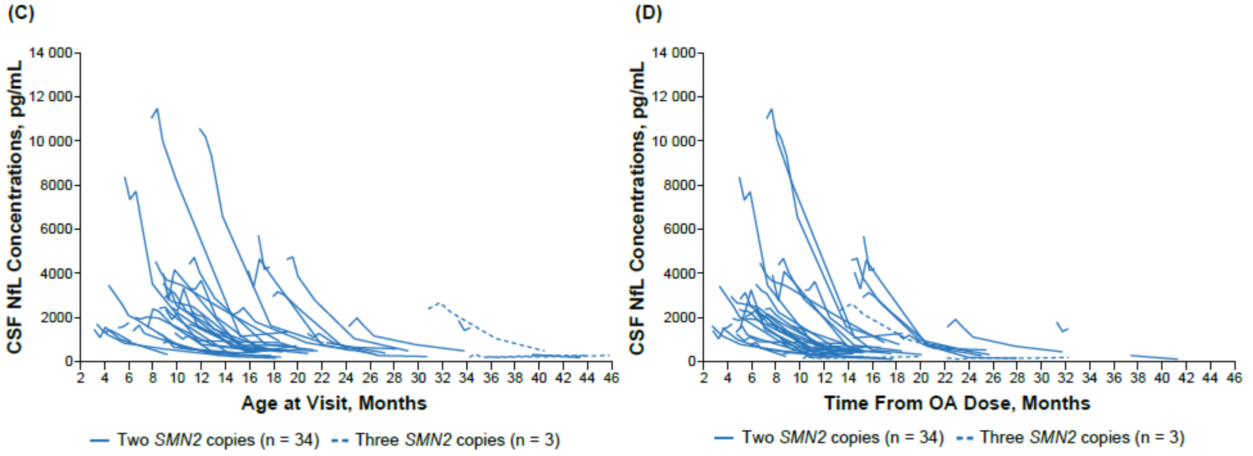

Individual participant trajectories may overlap. Descriptive statistics are not shown for participants with three *SMN2* copies due to small sample size. [a]Mean (SD) changes were calculated for participants who had assessments at each time point (baseline, Day 183 or Day 302) at the time of the NfL data-cut.

CSF = cerebrospinal fluid; NfL = neurofilament light chain; OA = onasemnogene abeparvovec; SE = standard error; SD = standard deviation; *SMN2* = *survival motor neuron 2*.
